# Substation equipment temperature prediction based on multivariate information fusion and deep learning network

**DOI:** 10.7717/peerj-cs.1172

**Published:** 2022-12-12

**Authors:** Lijie Sun, Chunxue Liu, Ying Wang, Zhaohong Bing

**Affiliations:** 1 School of Electronics and Information Engineering, Taizhou University, Taizhou, Zhejiang, China; 2 School of Information, Liaoning University, Shenyang, Liaoning, China; 3 Economic and Technological Research Institute of State Grid Heilongjiang Electric Power Co., Ltd., Haerbin, Heilongjiang, China; 4 Computing Technology Institute of East China, Shanghai, China

**Keywords:** Time series, CNN, GRU, Information fusion, PCA, Temperature prediction

## Abstract

**Background:**

Substation equipment temperature is difficult to achieve accurate prediction because of its typical seasonality, periodicity and instability, complex working environment and less available characteristic information.

**Methods:**

To overcome these difficulties, a substation equipment temperature prediction method is proposed based on multivariate information fusion, convolutional neural network (CNN) and gated recurrent unite (GRU) in this article. Firstly, according to the correlation analysis including linear correlation mapping, autocorrelation function and partial autocorrelation function for substation equipment temperature data, the feature vectors from ambient, time and space are determined, that is the multivariate information fusion feature vector (denoted as MIFFV); secondly, the dimension of MIFFV is reduced by principal component analysis (PCA), extract some of the most important features and form the reduced feature vector (denoted as RFV); then, CNN is used for deep learning to extract the relationship between RFV and the high-dimensional space feature, and construct the high-dimensional feature vector of multivariate time series (denoted as HDFV); finally, the high-dimensional feature vector is used to train GRU deep learning network and predict the equipment temperature.

**Results:**

A substation equipment in Taizhou City, Zhejiang Province is conducted by the method proposed in this article. Through the comparative experiment from the two aspects of features and methods, under the two prediction performance evaluation indexes of mean absolute percentage error (MAPE) and root mean square error (RSME), two main conclusions are drawn: (1) MIFFV from three aspects of ambient features, time features and space features have better prediction performance than the single feature vector and the combined feature vector of two aspects; (2) compared with other four related models under the same conditions, RFV is regarded as the input of the models, the proposed model has better prediction performance.

## Introduction

The safe operation of power equipment is the focus and key to ensure the stable operation of substation, in which substation primary equipment is the top priority; therefore, we should attach great importance to the primary equipment of the substation, strengthen management and control, and do a good job in the daily condition monitoring and maintenance of the primary equipment of the substation (Wang, 2016). Equipment temperature is an important index to measure the health of equipment, however, online monitoring is mainly for primary equipment (Sun, 2019), and many factors will cause the equipment temperature to rise, such as too much voltage load, an insufficiently tightened joint connection, loose bolts at key points, oxidized and corrode conductor surface, too much contact resistance of the contact surface, and so on. If the temperature rises slightly, the relevant electrical equipment will be damaged and burned, which will lead to the operation failure of the substation; more importantly, it will lead to fire and safety accidents, resulting in huge economic losses and social impact of the substation. Therefore, it is very important to know the temperature of each equipment in real time.

In the past, the substation was inspected and measured regularly by manual means which is prone to casualties, and in recent years, the state grid has adopted the intelligent inspection means for the management and monitoring of substation equipment, and installed infrared cameras in the substation, but due to the limited storage space of the equipment, it is generally set for one day or one hour, so sometimes the fault can not be found in time. Through substation equipment temperature prediction, the future temperature information is obtained in advance, and the purpose of equipment fault early warning can be realized.

When the data source and data set have been identified, the completion of equipment temperature prediction task mainly needs to go through two processes: feature engineering and modeling. This article focuses on these two links to solve the problem of accurate prediction of substation temperature.

Feature engineering mainly carries out feature selection and feature extraction. For substation equipment temperature prediction, in addition to the complex working environment of substation equipment, the biggest difficulty is that the information source used for prediction is limited. The research results in this field at home and abroad show that there are more domestic research results and less foreign research results. The research results are mainly concentrated in domestic Huazhong University of Science and Technology, Harbin University of technology, Zhejiang University, North China Electric Power University and some power companies (Hao et al., 2021; Guo et al., 2020; Kong, 2015). The research objects of substation equipment at home and abroad mainly include high-voltage or low-voltage switchgear (Velásquez, Lara & Melgar, 2019; Zeng et al., 2018; Bussière et al., 2017), intelligent electronic equipment (Sun et al., 2022), disconnector (Huang et al., 2022a), bushing contact (Huang et al., 2022b), *etc*. At present, most studies used historical time series as feature extraction source for rolling prediction of equipment temperature, which typically include auto-regressive and moving average model (ARMA) series models (AR, ARMA, ARIMA) (Baptista et al., 2018); however, the simple temperature trend can not accurately predict the future equipment temperature value, resulting in the failure to accurately identify the health status of the equipment and take precautions in advance. Some scholars are also constantly trying to find more feature sources. Through the seasonal analysis of substation equipment temperature data, it was found that there exists typical positive correlation between ambient temperature and equipment temperature. Therefore, the daily maximum temperature and daily minimum temperature are taken as ambient characteristics and equipment temperature at historical time to form a feature vector for equipment temperature prediction (Yu et al., 2022); in addition, by analyzing the influencing factors of temperature rise of high-voltage switchgear, Xu, Xu & He (2016) established a temperature prediction fusion model based on load current and ambient temperature of high-voltage switchgear by using information fusion technology and back propagation neural networBPNNk, and achieved good prediction performance. As is known, for primary equipment of substation main transformer, load current and equipment monitoring belong to different departments, so it is difficult to obtain load current information, and the daily maximum temperature and daily minimum temperature of the ambient can not clearly reflect the real-time correlation between the weather temperature and the equipment temperature, which will affect the prediction performance. Temperature is a parameter with heat transfer characteristics, and the temperature of adjacent positions in space has the effect of interaction. Based on current research, it can be seen that the traditional substation equipment temperature prediction method ignores the spatial relationship information of equipment in the historical time, resulting in poor prediction accuracy. Thus, it is particularly important to select what characteristics to characterize the temperature for prediction. So, when solving the problem of substation equipment temperature prediction, inspired by considering environmental perspective factor in the research results of the literature (Hou et al., 2021a), Feature extraction information comes from three viewpoints of ambient, time and space, and develops ambient feature vector, time feature vector and space feature vector as multivariate information fusion feature vector in this article. Considering that Zhejiang Province is a typical subtropical seasonal climate, the real-time weather temperature and humidity are selected as the ambient characteristics to form the ambient feature vector; the historical temperature time series of the monitoring points of the prediction target is selected as the time feature vector and the temperature of all monitoring points with space correlation for the predicted target monitoring point temperature is composed of space feature vector. Principal component analysis (PCA) (Zhang et al., 2022) is a common data analysis method and a linear dimensionality reduction method, whose principle is to map high-dimensional data to low-dimensional space through a certain linear projection, and expect the maximum amount of information (the largest variance) of the data on the projected dimension, so as to use fewer data dimensions and retain the characteristics of more original data points, which can be used to extract the main feature components of data. PCA has the functions of simplifying operation, removing data noise and discovering hidden related variables (Dai, 2021; Song & Yang, 2022), and it is adopted to reduce the feature vector of multivariate information fusion to form the reduced feature vector, so as to realize the feature extraction process for substation equipment temperature prediction.

The quality of the prediction model is also the main factor affecting the prediction performance. In the last five years, neural networks have been widely used in substation equipment temperature prediction, such as back propagation neural network (Liu, 2012), radial basis function neural network (Wang et al., 2015), generalized regression neural network (Kong & Zhang, 2016), adaptive neural network (Wang, 2015), neural network optimized by swarm intelligence algorithm (Xu, Hao & Zheng, 2020), support vector machine (SVM) and a series of other machine learning methods (Zhang et al., 2020). In the past three years, deep learning networks have made breakthrough, such as pedestrian trajectoryprediction (Esfahani, Song & Christensen, 2020), PM2.5 prediction (Mohammadshirazi et al., 2022), traffic speed prediction (Zheng, Chai & Katos, 2022), estimation of residual capacity for lithium-ion battery (Hou et al., 2022) and so on (Xu, Lin & Zhu, 2020). In 2021, Hou et al. (2021b) solved the problem of temperature prediction of switchgear equipment in substation by using long short-term memory (LSTM) network, and achieved good results, which opens the prelude of solving the problem of substation equipment temperature prediction with deep learning network. The gated recurrent unit (GRU) was proposed by Gharehbaghi et al. (2022) and is an effective variant of LSTM (Cao, Jiang & Gao, 2021; Yuan et al., 2022). In many cases, GRU and LSTM have the same excellent results, but GRU has fewer parameters, so it is relatively easy to train and the over fitting problem is lighter (Cao, Jiang & Gao, 2021; Yuan et al., 2022). Therefore, GRU network is adopt to predict substation equipment temperature in this article. Before the prediction, taking advantage of CNN’s feature extraction (Khalifani et al., 2022), CNN network is used for deep learning to extract the relationship between the reduced feature vector and the equipment temperature in the high-dimensional space, and construct the high-dimensional feature vector of multivariate time series, then the high-dimensional feature vector is used to train GRU network and predict the equipment temperature.

## Related Work

### Correlation analysis

Two functions of autocorrelation function and partial autocorrelation function are adopted to analyze correlation. The autocorrelation functon and partial autocorrelation function are described as follows. (1) As is known, autocorrelation belongs to sequence correlation, which expresses the cross-correlation between the sequence and itself at different moments (Chachlakis et al., 2021). The autocorrelation coefficient of the time series is denoted as ACF, that is autocorrelation function. This article quantitatively describes the lag autocorrelation of substation equipment temperature time series by calculating ACF value. ACF is expressed as ${\rho }_{k}^{\wedge }$ in formula Eq. (1):

(1) \begin{eqnarray*}{\rho }_{k}^{\wedge }= \frac{\sum _{t=1}^{n-k} \left( {Z}_{t}-Z^{-} \right) \left( {Z}_{t+k}-Z^{-} \right) }{\sum _{t=1}^{n}{ \left( {Z}_{t}-Z^{-} \right) }^{2}} \end{eqnarray*}

where, *Z*_*t*_ is the equipment temperature at time *t*, *Z*_*t*+*k*_ is the equipment temperature at time *t* + *k*, $Z^{-}$ is the average value of equipment temperature. (2) Partial autocorrelation is the relationship summary between the time series observation after eliminating interference and the previous time step observation (Mestre et al., 2021). That is, consider the correlation after removing the influence of intervention variables *Z*_*t*+1_, *Z*_*t*+2_, *Z*_*t*+3_, … with common linear dependence from *Z*_*t*_ and *Z*_*t*+*k*_, namely, under the condition of observation *Z*_*t*+1_, the autocorrelation state of *Z*_*t*_ and *Z*_*t*+*k*_ so on. Partial autocorrelation function (PACF) is expressed as *P*_*k*_ in formula Eq. (2):

(2) \begin{eqnarray*}{P}_{k}= \frac{Cov \left[ \left( {Z}_{t}-{Z}_{t}^{\wedge } \right) , \left( {Z}_{t+k}-{Z}_{t+k}^{\wedge } \right) \right] }{\sqrt{Var \left( {Z}_{t}-{Z}_{t}^{\wedge } \right) }\sqrt{Var \left( {Z}_{t+k}-{Z}_{t+k}^{\wedge } \right) }} \end{eqnarray*}

where, *Cov* refers to the covariance at moment *t*, *Var* refers to sample variance, ${Z}_{t}^{\wedge }$ is sample estimation at moment *t*, and ${Z}_{t+k}^{\wedge }$ is sample estimation at moment *t* + *k*.

### PCA

Principal component analysis(PCA) is a data dimension reduction method that is widely applied in various fields (Cao, Sun & Zhao, 2022), which has the functions of simplifying operation, removing data noise and discovering hidden related variables. Therefore, PCA is selected to screen the input features. By calculating cumulative contribution rate of the input features, the first few important features are selected from multiple features as the principal components to reduce the input dimension and improve the convergence speed.

The main idea of PCA is to relinearly combine p-dimensional linearly related features and map them into k-dimensional linearly independent features (*k* < *p*). The reacquired k-dimensional features are principal components, which can represent the information of the original features to the greatest extent.

It is assumed that it has *p*features, and each feature has *n* observation values, then the initial data matrix *C* can be obtained.

(3) \begin{eqnarray*}C= \left[ \begin{array}{@{}l@{}} \displaystyle {c}_{11}{c}_{12}\cdots {c}_{1p}\\ \displaystyle {c}_{21}{c}_{22}\cdots {c}_{2p}\\ \displaystyle \vdots \vdots \vdots \vdots \\ \displaystyle {c}_{n1}{c}_{n2}\cdots {c}_{np}. \end{array} \right] \end{eqnarray*}

The implementation process of PAC method is realized by the following six steps:

(1) The original *p* characteristics are standardized to obtain the standardized feature variables.

(4) \begin{eqnarray*}{y}_{j}= \frac{{s}_{j}-{\mu }_{j}}{{s}_{j}} ,j=1,2,\ldots ,p\end{eqnarray*}

where, ${\mu }_{j}= \frac{1}{n} {\mathop{\sum }\nolimits }_{i=1}^{n}{c}_{ij},{s}_{j}=\sqrt{ \frac{1}{n} {\mathop{\sum }\nolimits }_{i=1}^{n}{ \left( {c}_{ij}-{\mu }_{j} \right) }^{2}}$.

(2) Standardize each feature element to obtain the corresponding data matrix *W*.

(5) \begin{eqnarray*}W= \left[ \begin{array}{@{}l@{}} \displaystyle {w}_{11}{w}_{12}\cdots {w}_{1p}\\ \displaystyle {w}_{21}{w}_{22}\cdots {w}_{2p}\\ \displaystyle \vdots \vdots \vdots \vdots \\ \displaystyle {w}_{n1}{w}_{n2}\cdots {w}_{np} \end{array} \right] \end{eqnarray*}

where, ${w}_{ij}= \frac{{c}_{ij}-{\mu }_{ij}}{{s}_{j}} ,$i =1 , 2, …, *n*; j =1 , 2, …, *p*.

(3) According to the matrix *W*, the correlation coefficient matrix **$R={ \left( {r}_{ij} \right) }_{p\times p}$**of *W* is calculated. Where, ${r}_{ij}= \frac{{\mathop{\sum }\nolimits }_{t=1}^{n}{w}_{t1}{w}_{tj}}{n-1} ,i,$*j* =*1* , 2, …, *p*.

(4) Calculate the eigenvalues of matrix *R* andsort them in descending order *λ*_1_ ≥ *λ*_2_ ≥ ⋯ ≥ *λ*_*p*_, and the standard orthogonalization eigenvector corresponding to each eigenvalue is calculated *u*_1_, *u*_2_, …, *u*_*p*_, where, ${\mu }_{j}={ \left[ {\mu }_{1j},{\mu }_{2j},\ldots ,{\mu }_{pj} \right] }^{T},$j =1 , 2, …, *p*.

(5) *p* new feature vectors are computed with the original *p* standard orthogonal feature elements, that is,

(6) \begin{eqnarray*} \left\{ \begin{array}{@{}l@{}} \displaystyle {N}_{1}={u}_{11}{y}_{1}+{u}_{21}{y}_{2}+\cdots +{u}_{p1}{y}_{p}\\ \displaystyle {N}_{2}={u}_{12}{y}_{1}+{u}_{22}{y}_{2}+\cdots +{u}_{p2}{y}_{p}\\ \displaystyle \vdots \\ \displaystyle {N}_{p}={u}_{1p}{y}_{1}+{u}_{2p}{y}_{2}+\cdots +{u}_{pp}{y}_{p} \end{array} \right. \end{eqnarray*}

where, *N*_1_ refers to the first principal component; *N*_2_ is the second principal component; *N*_*p*_ is the *p* − *th* principal component.

(6) The contribution rate and cumulative contribution rate of each principal component are calculated, and the calculation formula is shown in formula Eq. (7) and formula Eq. (8) respectively.

(7) \begin{eqnarray*}{N}_{j}= \frac{{\lambda }_{j}}{\sum _{t=1}^{p}{\lambda }_{t}} ,j=1,2,\ldots ,p\end{eqnarray*}

(8) \begin{eqnarray*}{\eta }_{i}= \frac{\sum _{t=1}^{i}{\lambda }_{t}}{\sum _{t=1}^{p}{\lambda }_{t}} \times 100\text{%},i=1,2,\ldots ,p.\end{eqnarray*}

Among them, *N*_*j*_ is the contribution rate of the *j* − *th* principal component; *η*_*i*_ is the cumulative contribution rate of the first *i* principal components.

### CNN

**Figure 1:**
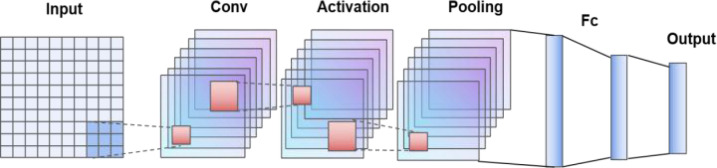
CNN model structure diagram.

CNN is the abbreviation of convolutional neural network, which is a variant of multilayer perceptron (MLP), and it was developed by biologists Huber and Wiesel in their early research on cat visual cortex (Aslan et al., 2022). Figure 1 shows the structure of CNN networks. The structure of CNN is described in order, including input layer, convolution layer, activation layer, pool layer, full connection layer and output layer. The convolution layer is the core structure of CNN model, which is usually 1 × 1 matrix, 3 × 3 matrix and 5 × 5 matrix. The weights of neurons on the same feature mapping plane in CNN can be shared locally. Therefore, CNN network supports parallel learning, which can greatly improve the calculation speed and model prediction efficiency. The unique structure of CNN has great advantages in the fields of machine learning, deep learning and prediction field, which is the most widely used depth feature extraction method.

### GRU network

**Figure 2:**
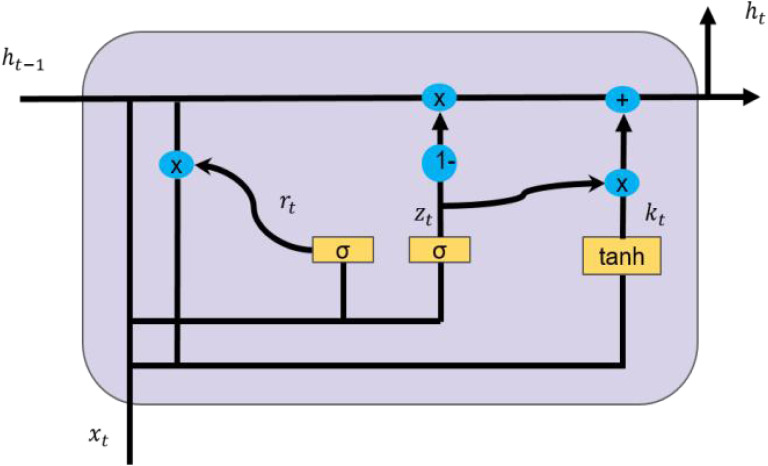
GRU structure diagram.

Gate recurrent unit (GRU) is a special network structure in neural network (Ansari, Bartoš & Lee, 2022), which has only two gate structures of reset gate and update gate, is simpler than the three gate structure of LSTM network and has good prediction effect. These two gating vectors can determine which data can be used as the final output. The basic structure of GRU is shown in Fig. 2.

In Fig. 2, *x*_*t*_ refers to the input data, that is, the high-dimensional feature vector, *h*_*t*−1_ refers to the output data of the previous layer, and *h*_*t*_ refers to the output data of the current layer. *r*_*t*_ and *z*_*t*_ are the outputs of reset gate and update gate, and *k*_*t*_ is the candidate set. *σ* and tanh are *sigmoid* activation function and tanh activation function. The mathematical description of GRU is shown in formula Eq. (9).

(9) \begin{eqnarray*} \left\{ \begin{array}{@{}l@{}} \displaystyle {z}_{t}=\sigma \left( {W}_{z}\cdot \left[ {h}_{t-1},{x}_{t} \right] +{b}_{z} \right) \\ \displaystyle {r}_{t}=\sigma \left( {W}_{r}\cdot \left[ {h}_{t-1},{x}_{t} \right] +{b}_{r} \right) \\ \displaystyle {k}_{t}=\tanh \nolimits \left( {W}_{k}\cdot \left[ {r}_{t}\cdot {h}_{t-1},{x}_{t} \right] +{b}_{k} \right) \\ \displaystyle {h}_{t}= \left( 1-{z}_{t} \right) \cdot {h}_{t-1}+{z}_{t}\cdot {k}_{t} \end{array}. \right. \end{eqnarray*}

## The Proposed Method

The substation equipment temperature prediction method proposed in this article is mainly realized by the following five steps:

(1) Correlation analysis. Linear graph correlation, autocorrelation and partial autocorrelation analysis are carried out for the temperature data of substation equipment;

(2) Determine the feature vector of multivariate information fusion. In this article, it includes the features from three aspects of ambient, time and space, which is denoted as *MIFFV*.

(3) Obtain the reduced feature vector. PCA is applied to reduce the dimension of multivariate information fusion feature vector to obtain the reduced feature vector, which denoted as *RFV*;

(4) CNN is used to extract the relationship between the reduced feature vector and the equipment temperature in the high-dimensional space, and construct the high-dimensional feature vector of multivariate time series, which is denoted as *HDFV*;

(5) *HDFV* is used to train GRU deep learning network and predict the equipment temperature.

**Figure 3:**
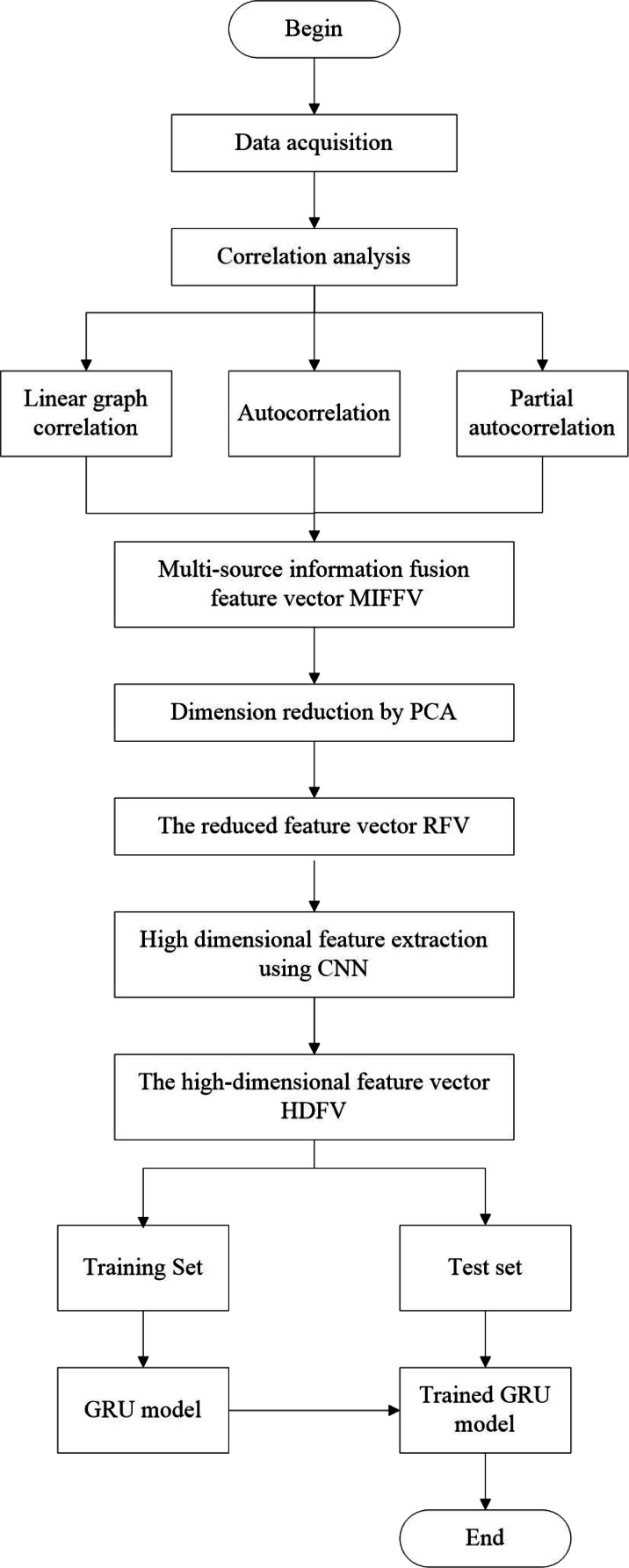
Flow chart of proposed method.

Flow chart of proposed method is shown in Fig. 3.

## Experiments and result analysis

### Temperature data acquisition of substation equipment

The research object of this article is primary equipment of main transformer in a substation from Taizhou City, Zhejiang Province. The substation adopts the intelligent inspection system. The temperature of each monitoring point for the equipment is measured by the infrared camera and stored in the form of multi-dimensional intelligent inspection history curve analysis report, including the substation equipment temperature monitoring serial number, organization, measurement position, inspection time, measured value and description (describe the equipment status, whether it is normal or not).

Substation equipment monitoring points are distributed at 110 kV side and 220 kV side, which includes four parts of bushing, conservator, heat sink and panorama. In this article, the data at 220 kV side are selected for the experiment. Monitoring point information is shown in Table 1.

**Table 1:** Monitoring point information table.

Serial number	Monitoring point name
1	220 kV bushing phase A contact
2	220 kV bushing phase B contact
3	220 kV bushing phase C contact
4	Conservator on 220 side
5	No. 1 heat sink on 220 side
6	No. 2 heat sink on 220 side
7	220 side equipment panorama

The infrared camera of the equipment is set to monitor once every hour, and the data acquisition time is 15 months from December 11, 2020 to March 10, 2022. However, there are power outage maintenance and bad points in the monitoring process. Therefore, this article adopts the method of direct elimination, and finally obtains 3,906 effective experimental data.

The selection of data will directly affect the effectiveness of the prediction model. According to the typical seasonal characteristics of temperature, this article selects the data of the first 12 months in the experimental data for training, that is, 3,086 data from December 11, 2020 to December 10, 2021, and 820 data from December 11, 2021 to March 10, 2022.

**Figure 4:**
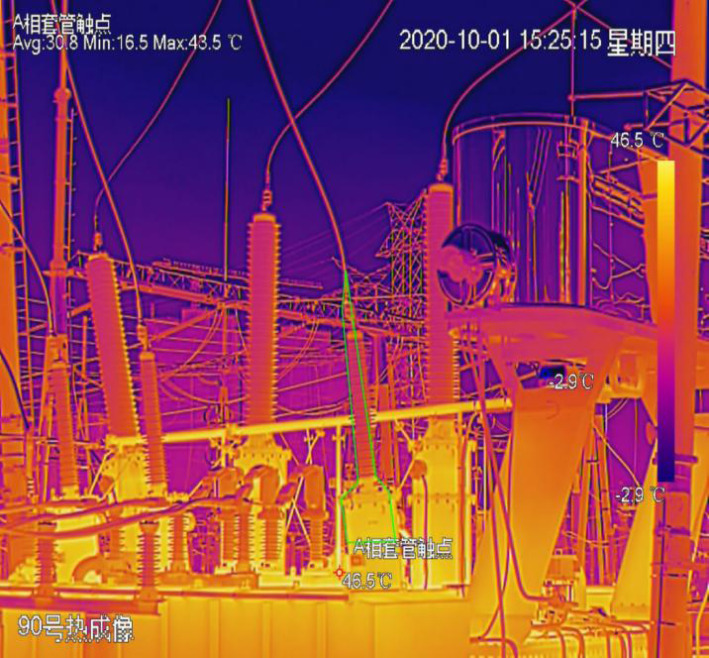
Thermal imaging of phase a contact at 220 kV side bushing on October 1, 2020.

For the primary equipment of main transformer in substation, the temperature of bushing has the greatest impact on the equipment, so the temperature of A contact from bushing phase is selected as the prediction target for the experiment. Figure 4 shows the thermal imaging diagram of phase A contact at 220kV side bushing on October 1, 2020.

### Feature engineering

#### A. Data Analysis and Feature Selection

**Figure 5:**
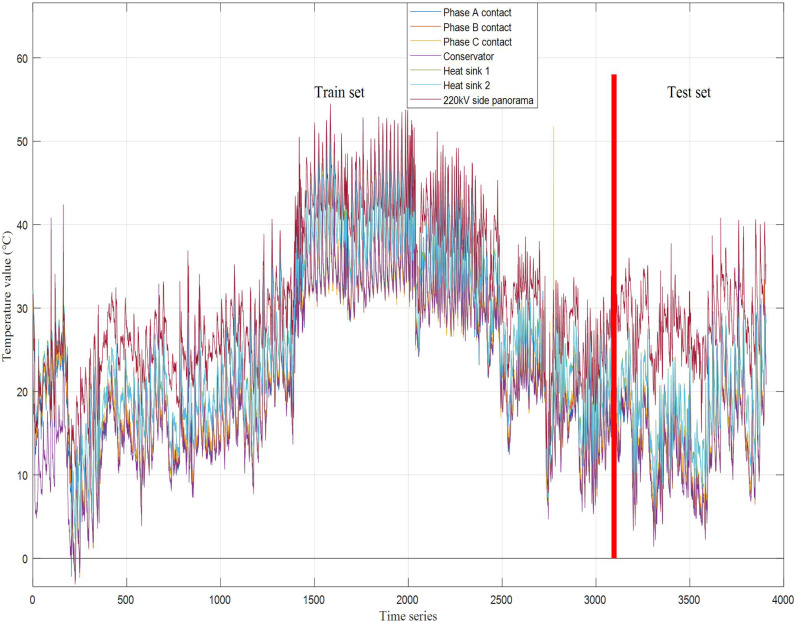
Temperature trend diagram of all monitoring points on 220 kV side.

Figure 5 shows the temperature data of all monitoring points on 220 kV side from the primary equipment of No. 2 main transformer, and two conclusions can be drawn: (1) With seasonal changes, the equipment temperature also changes significantly. The corresponding performance of the same monitoring point in different seasons is different. The average temperature in winter is about 20 °C and the average temperature in summer is about 50 °C. It can be seen that there is obvious correlation between equipment temperature and environmental factors. Therefore, when predicting the equipment temperature, it is necessary to consider the ambient temperature factor. (2) The temperature trend of different monitoring points for the same equipment shows obvious consistency, which means that there is typical linear correlation between the temperature of equipment space correlation monitoring points.points for the same equipment shows obvious consistency, which means that there is typical linear correlation between the temperature of equipment space correlation monitoring points.

**Figure 6:**
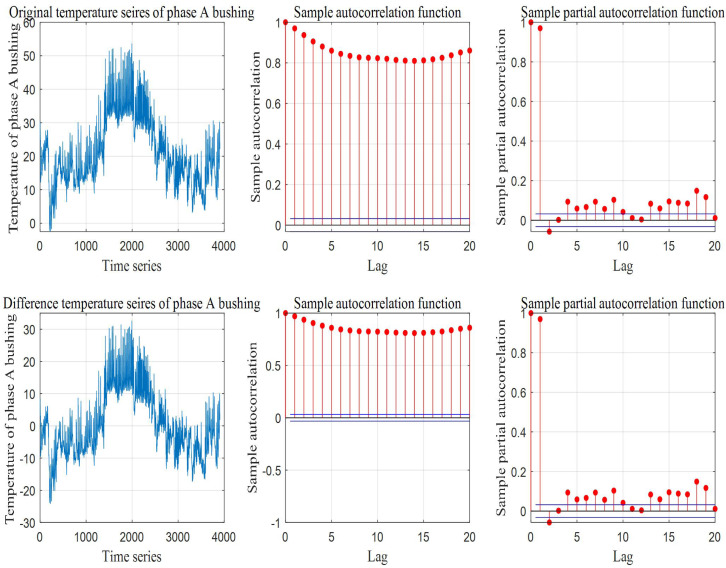
ACF and PACF analysis of equipment temperature time series.

In addition, Fig. 6 shows the correlation analysis results of historical temperature time series from phase A contact of bushing. According to the analysis results of autocorrelation and partial autocorrelation, it can be determined that the temperature time series of phase A contact for bushing is an unstable series. From ACF and PACF between the temperature time series and its first-order difference series, it can be seen that they are trailing, indicating that the historical temperature of substation equipment has strong correlation, and the influence of past time decreases gradually with the passage of time.

In summary, the substation equipment temperature has typical seasonality, periodicity and instability. Therefore, when predicting the equipment temperature, this article determines that the feature vector of multivariate information fusion is composed of the characteristics of ambient, time and space, which is recorded as $MIFFV= \left[ A,T,S \right] $, where, *A* refers to the ambient feature, *T* refers to the time feature and *S*refers to the space feature. The specific description is as follows:

(1) Ambient feature. In Part A, it is found that the substation equipment temperature is greatly affected by the ambient temperature. Therefore, the weather conditions are taken as the ambient feature in this article, which are recorded as $A= \left[ {A}_{1},{A}_{2},{A}_{3},\ldots \ldots ,{A}_{d1} \right] $, *d*1 is the dimension of ambient feature. In addition, considering that Zhejiang Province belongs to a typical subtropical monsoon climate, with low temperature and little rain in winter, prevailing northwest wind, high temperature and rain in summer, prevailing southeast wind and muggy, this article determines to take real-time weather temperature and humidity as ambient characteristics to form the ambient feature vector($A= \left[ {A}_{1},{A}_{2} \right] $, that is, set *d*1 = 2). Because the temperature of substation equipment is set to be collected every hour, in order to obtain ambient characteristics, Java programming is used to collect weather conditions every hour through the weather interface of Juhe API (website: http://www.juhe.cn), and two columns of weather temperature and humidity are selected as ambient characteristics.
(2) Time feature. According to the working experience of substation operation and maintenance personnel and the autocorrelation and partial autocorrelation analysis results, the time series of substation equipment temperature has strong lag correlation. Therefore, the historical temperature time series of substation equipment is selected as the time feature vector, which is recorded as $T= \left[ {T}_{1},{T}_{2},\ldots \ldots ,{T}_{d2} \right] $. Although the lag correlation is relatively large, considering that this article adopts the feature vector of multi information fusion, in order to avoid the inclination of the feature vector in the time feature due to too many time features, the equipment temperature values of the past three times are selected as the time feature, that is *d*2 = 3.
(3) Space feature. The primary equipment of No. 2 main transformer is taken as the research object. For such substation equipment, including 110 kV and 220 kV sides, and both sides are relatively independent, the article selects the temperature of phase A contact for bushing on 220 kV side as the prediction target for the experiment. Therefore the temperature of all monitoring points with space correlation with phase A contact for bushing is composed of space feature vector, which is recorded as $S= \left[ {S}_{1},{S}_{2},\ldots \ldots ,{S}_{d3} \right] $. The names of all monitoring points are recorded in Table 1. There are seven infrared temperature monitoring points on the 220 kV side, that is, in addition to the bushing phase A contact, there are six spatial correlation monitoring points, namely bushing B-phase contact, bushing c-phase contact, conservator, No. 1 heat sink, No. 2 heat sink temperature and 220 kV side panoramic temperature. Therefore, set *d*3 = 6.

#### B. Feature extraction—reduced feature vector based on PCA

**Figure 7:**
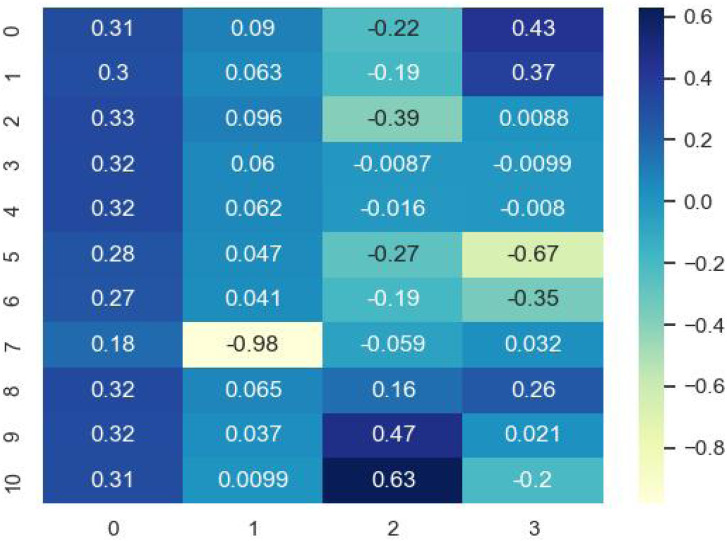
PCA dimensionality reduction mapping matrix.

**Figure 8:**
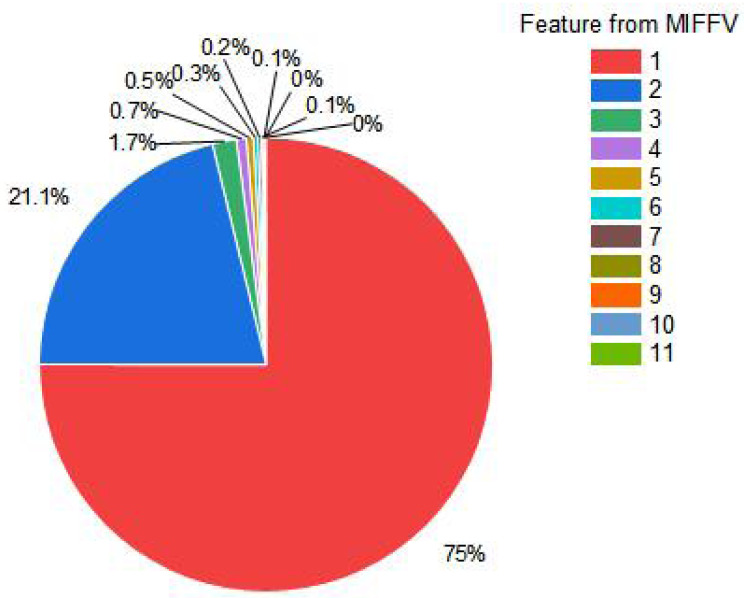
Feature contribution rate pie chart.

There are 11 characteristics in *MIFFV* of multivariate information fusion composed of three aspects of ambient, time and space, which can comprehensively characterize the temperature. While, too much input data can not improve prediction accuracy, but it is easier to produce information redundancy. Therefore, PCA is adopted to reduce the dimension. In general, the eigenvector composed of eigenvalues with cumulative contribution rate of 85%–95% is used as the principal component. Through many experiments, it is verified that the effect of the eigenvalue prediction is the best when the cumulative contribution rate reaches 98%, therefore, the principal components are taken as the reduced feature vector (denoted as *RFV*) under 98% cumulative contribution rate in this article. In the experimental process, PCA dimensionality reduction mapping matrix is shown in Fig. 7, and the feature contribution rate pie chart is shown in Fig. 8.

#### C. Feature extraction—depth feature mining based on CNN

Before establishing the prediction model, take advantage of CNN feature extraction, apply it to deep learning, and extract the relationship between reduced feature vector and equipment temperature in high-dimensional space; that is, the reduced feature vector *RFV* obtained by PCA is taken as input data of CNN model, *RFV* of low dimension is mapped to high dimension space, and the high-dimensional feature vector of multivariate time series is constructed, which is *HDFV*, and it is the output of the CNN model.

### Temperature prediction of substation equipment

Deep learning network based on CNN and GRU (CNN-GRU) is adopted to predict the phase A contact of bushing, where, CNN filter size is 10; the training cycle is 24 times per round, 60 rounds in total, and the total number of iterations is 1,440; the learning rate is 0.005 and the error threshold is 0.001.

**Figure 9:**
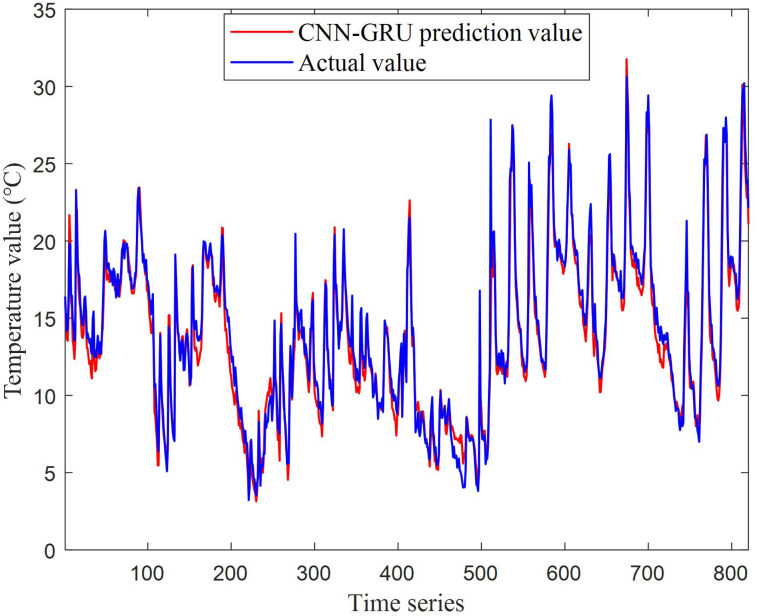
The prediction results for test set based on CNN-GRU.

**Figure 10:**
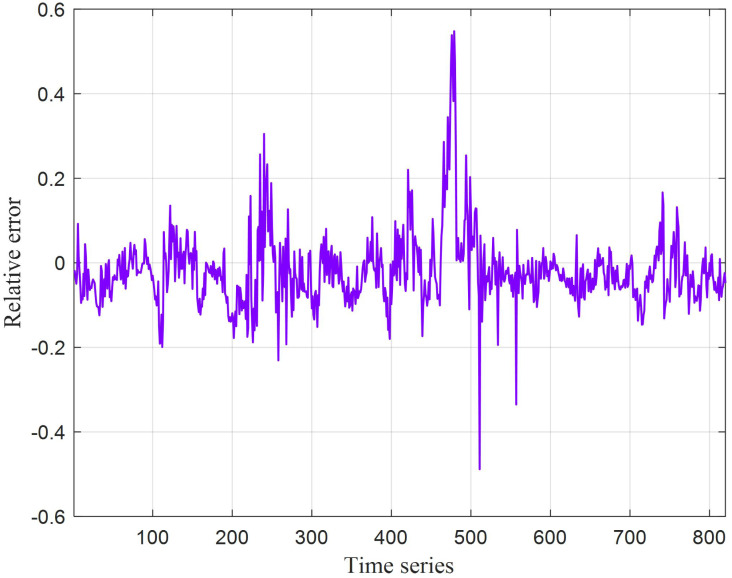
The testing relative error based on CNN-GRU.

The prediction results for test set based on CNN-GRU are shown in Fig. 9, and the testing relative error is shown in Fig. 9. From the above results, it can be summed up that the temperature prediction effect of bushing phase A contact based on CNN-GRU network is good, the relative error remains between ±0.2, and there is a relatively large error between the sample 450 and 500 in the test set from Fig. 10. The results show that because too many missing points and bad points are eliminated during this period, resulting in the model not obtaining a perfect model for a period of time. In the future, when dealing with missing points, it can be considered using fuzzy c-means clustering and other methods to complete the data to improve the prediction performance of the model.

## Prediction Performance Test and Result Analysis

### Evaluation index of predictive performance

(1) MAPE

MAPE refers to mean absolute percentage error, which is expressed by the formula Eq. (10):

(10) \begin{eqnarray*}MAPE= \frac{100\text{%}}{n} \sum _{i=1}^{n} \left\vert \frac{{y^{\wedge }}_{i}-{y}_{i}}{{y}_{i}} \right\vert \end{eqnarray*}

where, *y*_*i*_ is true value of equipment temperature, and ${y^{\wedge }}_{i}$ is the predicted value of equipment temperature. The range of MAPE belongs to $ \left( 0,+\infty \right) $, MAPE value of 0% means perfect model, and MAPE value greater than 100% indicates relatively poor model. (2) RMSE

RMSE refers to root mean square error, which is expressed by the formula Eq. (11):

(11) \begin{eqnarray*}RMSE=\sqrt{ \frac{1}{\mathrm{n}} \sum _{i=1}^{n}{ \left( {y}_{i}^{\wedge }-{y}_{i} \right) }^{2}}\end{eqnarray*}

where, *y*_*i*_ and ${y^{\wedge }}_{i}$ means the same with the formula (10); the range of RMSE is $ \left( 0,+\infty \right) $, and the error is positively correlated with RMSE value. When the predicted value is exactly the same as the actual value, it is equal to 0.

### Comparative experiments

Aiming at verifying the effectiveness of this method, comparative experiments from two aspects are carried out in this article:

(1) The prediction performance under different characteristics is compared. Comparative features include only time feature *T*, only ambient feature *A*, only spatial feature *S*, multivariate information fusion feature vector *MIFFV* andthe reduced feature vector *RFV*, and CNN-GRU network is adopt as the prediction model to predict the temperature of phase A contact. The comparison results are listed in Table 2.

**Table 2:** Prediction performance comparison of CNN-GRU network under different feature vectors.

Feature vector	MAPE	RMSE
A	19.65	244.84
T	12.58	212.14
S	18.52	245.05
MIFFV	6.98	122.12
RFV (MIFFV+PCA)	5.48	95.54

(2) The prediction performance of different models is compared under the same conditions. The reduced feature vector *RFV* proposed in this article is taken as the input data, and CNN-GRU network is compared with four other network models of BPNN (back propagation neural network), WaveNN (wavelet neural network, in which the Morlet wavelet is adopt), LSTM (long short term networks) and CNN-LSTM. During the comparative experiments, the parameters such as iteration times, learning rate and error threshold are the same in all prediction models. Prediction results of different models are compared in Table 3.

**Table 3:** Prediction performance comparison results of different models.

Model	MAPE	RMSE
BPNN	5.98	101.47
WaveNN (morlet)	7.93	120.93
LSTM	6.50	131.55
CNN-LSTM	6.26	100.17
CNN-GRU	5.48	95.54

### Analysis of prediction results

According to the above comparative experiments, this article analyzes the prediction results from multiple angles and draws the following conclusions from the statistical results from Tables 2 and 3:

(1) CNN-GRU was applied to the prediction performance comparison experiment under different feature conditions, and the results showed that the multi-source information fusion feature vector constructed from the three aspects of ambient, time and space is better than the single feature prediction effect, in which MAPE and RMSE were reduced by one order of magnitude; that is, *MIFFV* includes rich information than the *A*, *T* and *S* feature;
(2) The reduced feature vector *RFV* composed of principal components extracted after PCA dimensionality reduction had better prediction performance than *MIFFV* (MAPE is decreased from 6.98 to 5.48, and RMSE is decreased from 122.12 to 95.54), which shows that feature extraction plays a significant role in the prediction process, and the feature engineering scheme proposed in this article has the best effect on the temperature prediction of substation equipment.
(3) Compared with CNN-LSTM, CNN-GRU had better performance, which shows that although GRU with two gating structures are simpler than LSTM three gating structures, GRU has better effect in temperature prediction of substation equipment;
(4) CNN-LSTM had better effect than LSTM, which shows that CNN can mine the characteristics of equipment temperature depth when it is used for high-dimensional feature extraction, and provides a guarantee for the prediction model to achieve better prediction effect;
(5) The depth network models of LSTM, CNN-LSTM and CNN-GRU had better prediction effect than the shallow networks of BPNN and WaveNN shallow networks, which shows that the deep learning network has obvious advantages in the field of prediction compared with the shallow networks in traditional machine learning.

## Conclusions

In the process of substation equipment temperature prediction, the prediction effect is not ideal due to less information sources; the problem is solved from the two links of feature engineering and prediction modeling. In the aspect of feature engineering, linear graph correlation, autocorrelation and partial autocorrelation function analysis are applied to establish the feature vector of multi-source information fusion from the three aspects of environment, time and space. After PAC dimension reduction, the principal component is obtained as the reduced feature vector. Finally, the equipment temperature is predicted through CNN-GRU double-layer depth network model, in which CNN realizes depth feature extraction. The effectiveness of this method is fully proved by comparative experiments from two aspects of different feature vectors and different prediction models. However, in practice, it is usually necessary to obtain the equipment temperature at more times in advance, so the next goal is to realize the multi-step accurate prediction of substation equipment temperature.

##  Supplemental Information

10.7717/peerj-cs.1172/supp-1 Supplemental Information 1 Data and code of substation equipment temperature prediction based on multivariate information fusion and deep learning network

## Additional Information and Declarations

### Data Availability

The following information was supplied regarding data availability: The raw data and code are available in the Supplemental Files.
